# A Time-Division Multiplexing Multi-Channel Micro-Electrochemical Workstation with Carbon-Based Material Electrodes for Online L-Trosine Detection

**DOI:** 10.3390/s23146252

**Published:** 2023-07-09

**Authors:** Qiwen Bao, Gang Li, Zhengchun Yang, Jun Wei, Wenbo Cheng, Zilian Qu, Ling Lin

**Affiliations:** 1 School of Precision Instrument and Optoelectronic Engineering, the State Key Laboratory of Precision Measuring Technology and Instruments, Tianjin University, 92 Weijin Road, Tianjin 300072, China; baoqiwen@tju.edu.cn (Q.B.); ligang59@tju.edu.cn (G.L.); 2 School of Electrical and Electronic Engineering, Tianjin Key Laboratory of Film Electronic & Communication Devices, Advanced Materials and Printed Electronics Center, Tianjin University of Technology, Tianjin 300384, China; yangzhengchuntjut@163.com; 3 School of Materials Science and Engineering, Harbin Institute of Technology, Shenzhen 518055, China; junwei@hit.edu.cn; 4 Suzhou Institute of Biomedical Engineering and Technology, Chinese Academy of Sciences, Suzhou 215163, China; chengwb@sibet.ac.cn; 5 Beijing Information Technol Coll, Beijing 100015, China; zilian_qu@163.com

**Keywords:** multiple channel, time-division multiplexing, electrochemical working station, smartphone, CB-GO, L-tyrosine

## Abstract

In the background of the rapid development of artificial intelligence, big data, IoT, 5G/6G, and other technologies, electrochemical sensors pose higher requirements for high-throughput detection. In this study, we developed a workstation with up to 10 channels, which supports both parallel signal stimulation and online electrochemical analysis functions. The platform was wired to a highly integrated Bluetooth chip used for wireless data transmission and can be visualized on a smartphone. We used this electrochemical test platform with carbon–graphene oxide/screen-printed carbon electrodes (CB-GO/SPCE) for the online analysis of L-tyrosine (Tyr), and the electrochemical performance and stability of the electrodes were examined by differential pulse voltammetry (DPV). The CB-GO-based screen-printed array electrodes with a multichannel electrochemical platform for Tyr detection showed a low detection limit (20 μM), good interference immunity, and 10-day stability in the range of 20–200 μM. This convenient electrochemical analytical device enables high-throughput detection and has good economic benefits that can contribute to the improvement of the accuracy of electrochemical analysis and the popularization of electrochemical detection methods in a wide range of fields.

## 1. Introduction

Sensor information fusion has rapidly developed in the last decade due to the rapid development of computer and artificial intelligence technologies. It is a technique for acquiring multi-channel sensor data and performing intelligent fusion in a distributed sensor environment [1]. A good test system is similar to a human body with normal body functions. The human body senses light, odor, mechanical wave, taste, and temperature signals through the eyes, nose, ears, tongue, and skin, and fuses and outputs the information through input to the brain. A lot of inspiration from nature has been put into research and even application for the development of technology. Simulating the human body to create a biosensor-like device has become a challenge that many researchers in the field of sensors are currently working to overcome [2].

This fused sensor system consists of three components: data acquisition, data integration, and data transmission. Data acquisition relies mainly on various types of sensors, which can be classified according to their operating principles: magnetic sensors [3,4], optoelectronic sensors [5,6], semiconductor sensors [7,8], electrochemical sensors [9,10], among others. Among them, electrochemical sensors are extremely suitable for smart sensors based on big data due to their simple manufacturing process, wide range of applications, and low cost compared to traditional sensors.

The electrochemical sensor principle is based on the electrochemical properties of the substance to be measured and transforms the chemical quantity of the substance to be measured into electrical power for sensing. Hence, electrochemical sensors have a wide range of applications, such as food safety [11,12], biomedical devices [13,14], industrial production [15,16], and wearable devices [17,18]. At the same time, its simple principle leads to excessive noise and errors introduced into the system, where multiple known and unknown electrochemical reactions often occur simultaneously in the test system. Also, external environmental variables such as temperature and pH can affect the stability of the test system, and these factors will eventually be transferred to the end device in a series of electrical forms such as current, voltage, and resistance, leading to a decrease in the reliability and stability of the sensor. Shah et al. [19] designed and prepared a glassy carbon electrode based on L-tryptophan modification for trace analysis of As(III) considering the effect of pH on the electrode. Nithya et al. [20] designed an electrochemical system for the selective detection of NH_3_ using Ni^2+^-doped CuO as the anode and LSC55 as the cathode, and also studied the electrochemical properties of the electrode in the range of 300–650 °C and found that the CuO electrode harvested the highest sensitivity at 550 °C. It can be found that it is particularly important to design an electrode array integrating multiple channels for a more reliable electrochemical sensor.

With the rapid development of integrated circuits, the computing power of smartphones has been increasing. Most systems have large high-definition displays, easy-to-operate systems, Bluetooth, and NFC modules for wireless data transmission. Thus, researchers have increasingly focused on the development of portable, easy-to-operate, and powerful electrochemical workstations. With a communication distance of 10–100 m, Bluetooth technology has a very high data transmission rate, is suitable for complex testing environments, and meets real-time data transmission needs [21,22,23].

In this study, an electrochemical testing system was constructed in order to determine the stability, efficiency, and reliability of the testing electrode in the online detection and analysis of L-tyrosine. An electrode array composed of 10 electrodes was used to construct an electrochemical workstation that could be time-shared and reused. It supported up to nine working electrodes and one reference or counter-electrode at the same time. This array was used to test the stability of standard electrodes; the performance test of multiple electrodes was completed automatically by configuring a built-in program command to save time and reduce the accidental error caused by human operation. When used to test the specificity of a single electrode, the laboratory environment can be equipped with multiple electrochemical electrodes that can be used to test the interference and predict the level of biochemical substances in the test environment in advance. In the field, electrochemical equipment can be used to quantitatively measure the level of potential disruptors and account for these in the results.

More specifically, we constructed a Nafion macromolecule/carbon–graphene oxide/screen-printed carbon electrode (CB-GO/SPCE)-laminated electrode based on a screen-printed substrate and mounted it on our multi-channel electrochemical workstation for the real-time monitoring of L-tyrosine (Tyr). CB-GO has many advantages such as the requirement of a simple synthesis method [24], unique photoelectric characteristics [25], suitable biocompatibility [26], and excellent electrochemical performance [27].

## 2. Materials and Methods

### 2.1. Design of Time-Division Multiplexing (TDM) Portable Electrochemical Workstation System

**Figure 1:**
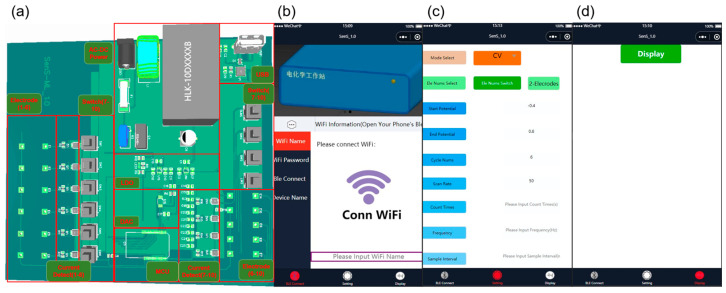
(**a**) System design of multi-channel portable electrochemical testing equipment. (**b**) Connection interface between the mobile app and device. (**c**) Mobile app configuration of working modes and operation parameter interface. (**d**) Device data display interface for mobile app Bluetooth acquisition.

Figure 1 shows the system block diagram and software interface for the whole device. An AC–DC voltage conversion module provided a voltage of +5 V to the circuits, and the power supply was provided by USB. This went through two routing voltage regulator output circuits (MIC520, Micrel, San Jose, CA, USA) to a digital voltage output and an analog voltage output. The control commands were set through an application on the smartphone, as shown in Figure 1c.

Through the app, the test method and parameters were set, such as cyclic voltammetry (CV), differential pulse voltammetry (DPV), square wave voltammetry (SWV), timing current method (CA), the electrode mode, start/stop voltage, number of cycles, scanning speed, counting time, frequency, sampling interval, potential, amplitude, pulse width, and pulse period width. The potentiostation was composed of a double-channel high-precision digital-to-analog converter (DAC) chip (AD5667RBRMZ) and its surrounding circuit. This was used to control the potential on the working electrode and the opposite electrode. The connection of each channel was controlled by a key switch, and the measured potential or current signal was coupled to the input port of the current detection chip (MAX9934FAUA+). The digital signal after analog–digital conversion could be displayed in real time on a 4.3-inch LCD, or transmitted to a smartphone through the on-chip antenna via the Bluetooth function of a microprogrammed control unit (MCU). The interface of the app and device connection and the data transmission on the mobile phone are shown in Figure 1b,d. A demonstration schematic diagram is shown in Figure S1. Table 1 shows the components used in the equipment and the unit price of bulk purchase. This portable multi-channel electrochemical analysis device was designed as a cost-effective alternative to commercial electrochemical workstations costing thousands of dollars.

### 2.2. Chemical and Apparatus

The CB (Carbon Vulcan XC-72) and GO solutions (mean radial dimension: 5–8 μm, thickness: 1 nm, concentration: 2 mg/mL, dispersed in H_2_O), Tyr (99%, 60-18-4, 25 g), sarcosine (99%, 25 g, 107-97-1), L-serine (99%, 100 g, 56-45-1), L-arginine (98%, 100 g, 74-79-3), L-threonine (99%, 100 g, 72-19-5), DL-alanine (99%, 100 g, 302-72-7), and N-dimethylformamide (DMF; AR, 99.5%, 500 mL) were purchased from Macklin Biochemical Co., Ltd (Shanghai, China). Ethyl alcohol and phosphate buffer (PBS) were purchased from Sigma Aldrich (St Louis, MO, USA). Ag/AgCl, CP, and Pt electrodes were purchased from Lanlike Chemical Electronic High Technology Co., Ltd (Tianjin, China). Nafion D520 dispersion liquid was purchased from Dupont Co., Ltd (Wilmington, DE, USA). Electrochemical measurements were performed at ambient temperature (20–25 °C). Deionized (DI) water (~18.0 MΩ) was obtained from a Millipore system.

### 2.3. Chemical Modification of the Nafion/CB-GO/SPCE

**Figure 2:**
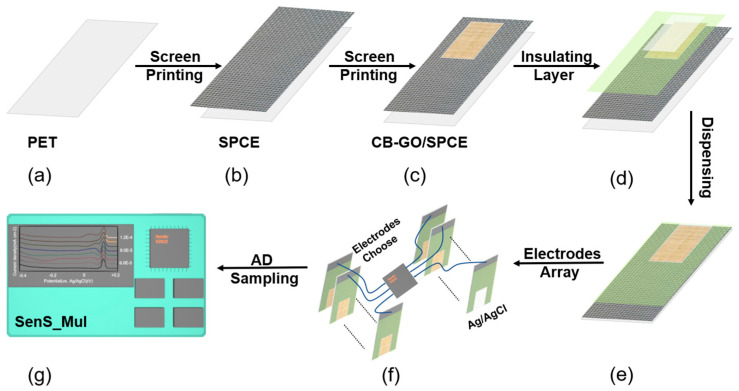
Electrode fabrication process and modification. (**a**) PET. (**b**) Screen-printed SPCE with a pure carbon substrate pattern. (**c**) Screen printing of CB-GO/SPCE of the active material CB-GO. (**d**) Silk-screened CB-GO/SPCE of the isolation layer. (**e**) Nafion/CB-GO/SPCE prepared by dispensing method. (**f**) A multi-channel array of mass-produced CB-GO/SPCE electrodes. (**g**) On-line testing using a portable electrochemical workstation.

Figure 2 illustrates the preparation process of Nafion/CB-GO/SPCE, which includes the micro-dispensing process of the layer-by-layer screen-printing process. It was necessary to design the specific layout pattern prior to screen printing, and then customize the corresponding screen. In this study, a long rectangular mesh for printing a pure carbon substrate structure (Figure 2b), small rectangular mesh for printing CB-GO active material (Figure 2c), and short rectangular mesh for printing the center openings of the insulation layer (Figure 2d) were designed and customized. The PET and mesh plates were cleaned with anhydrous ethanol and deionized water, then dried using a hot air dryer for 3 h at 60 °C. The printing of the thin-film materials was as follows: 5 mg of CB powder was added to 5 mL of 1 mg/mL of DMF solution, and sonicated for 1 h. A total of 3 mL of the dispersed CB solution and 4 mL of the GO dispersion solution were mixed and then ultrasonically dispersed for 30 min.

As seen in Figure 2a–e, the pure carbon substrate pattern for the conductive substrate layer was placed using an automatic flat screen-printing machine WY-4060 from Screen Printing Fest Screen Printing Materials Co., Ltd. (Langfang, China) with layer-by-layer printing. The CB-GO active electrode layer provided the electrochemical reaction area and insulating isolation layer. Next, Figure 2e shows a dispenser SM200SX-3A from MUSASHI (Tokyo, Japan, Adachi Ward) being used to draw the Nafion ethanol dispenser at the central opening and to uniformly draw a solid rectangular pattern for ion selective passage (power: 15.9 kW).

### 2.4. Software Design Process

**Figure 3:**
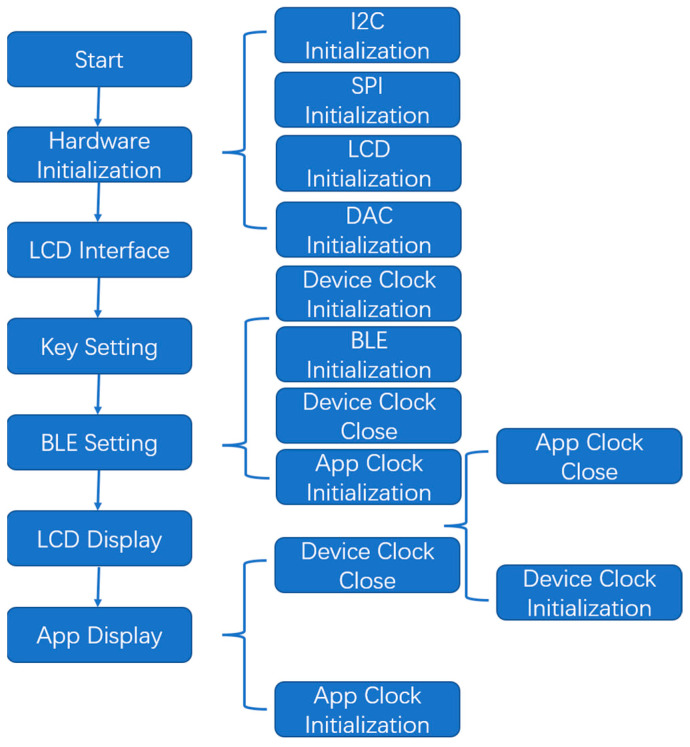
Main program design flow and Bluetooth information configuration.

As shown in Figure 3, the DAC communicates with the MCU through the I^2^C bus, while the LCD communicates with the MCU through a serial peripheral interface (SPI). Therefore, the hardware initialization of the program included transmit data (TXD) and receive data (RXD) pins, frequency, interrupt priority, and other parameters used in the configuration of the I^2^C initialization program. The SPI_CS, SPI_MOSI, and SPI_SCK pins were required for the SPI initialization program configuration. LCD initialization set the positive gamma control, power control, memory access control, and interface pixel format of the LCD through a series of control and display commands.

During the DAC initialization, the working mode (such as the reference voltage sources) of the two DAC channels was configured. The key-setting subroutine determined the working and electrode modes by pressing the keys. If the key setting was not set, the default working mode was selected. Bluetooth Low Energy (BLE) settings were used to set corresponding operating parameters through the app, such as the start/stop voltage, cycle times, scanning speed, timing period, frequency, and sampling interval. It is worth noting that if you choose the service uploaded by the app through a Bluetooth device to display the running results in real time, you need to turn off the clock switch of the device in advance, because the app clock conflicts with the device clock. Similarly, the LCD display subroutine also requires the app clock to be off when working.

### 2.5. The Workstation Equipped with the Nafion/CB-GO/SPCE for Tyr Detection

Firstly, PBS (0.01 M, pH = 7.2) was prepared by adding 300 mL of deionized water and buffer powder into a customized cylindrical electrolytic cell. The electrolytic cell was specially designed for the multi-channel electrochemical workstation and could be paired with up to 10 electrode columns (including the reference and counter-electrodes) at the same time. As shown in Figure S1, five working electrodes, one common reference electrode, and one counter-electrode were set up. When working, the key switch was used to switch on and off the corresponding working electrode channel, so that a performance test of five electrodes could be completed in sequence by adding the test substance.

As shown in Figure S1, the electrodes were connected to the micro-workstation. To make it as portable as possible, the external power supply was connected to the workstation by USB. At the same time, with the help of powerful smartphones, we could set the working mode, electrode mode, and operation parameters of the workstation through virtual keys and input boxes. Then, the compiled binary code was sent to the device through the Bluetooth service program, and it was assigned to a corresponding variable through a data format conversion function in the main program of the device. The value of the variable set by the clock parameters determines the specific patterns of timer handler; selecting double or triple electrode models determines the signal to switch on and off; the start/end voltage sets the DAC, which controls the initial potentials of two electrodes and the terminate potential; cycles determine the DAC scan times; and timing times back and forth to determine two die I-t/OP. The working time and frequency of the main program were set as the square wave frequency of the SWV mode, and the sampling interval was set as CV, DPV, LSV, data sampling width, and increase potential of the NPV mode. The increase potential of each pulse was set as the amplitude of the DPV mode, the pulse width was set as the potential pulse width, and the pulse period width was set as the potential pulse period or dropping of the NPV/DPV mode time.

To minimize error, the same electrodes were used for a set of experiments. During Tyr detection, the electrochemical performance of the five working electrodes was tested in sequence by adding Tyr powder weighed by a precision balance directly into the PBS solution. To accelerate the ion transfer rate and the full dissolution of Tyr powder, the reaction environment was placed on a magnetic stirrer with adjustable temperature and online pH detection. After the experimental group was tested, the Tyr concentration of the solution to be tested was displayed in real time through the LCD display or on the mobile app. The specificity and repeatability tests were conducted as described previously. The performance tests of the five electrodes were carried out in a one-time test, and the test data were sent to the mobile app through LCD display results and Bluetooth.

## 3. Results

### 3.1. Characterization of the Tyr Sensor

**Figure 4:**
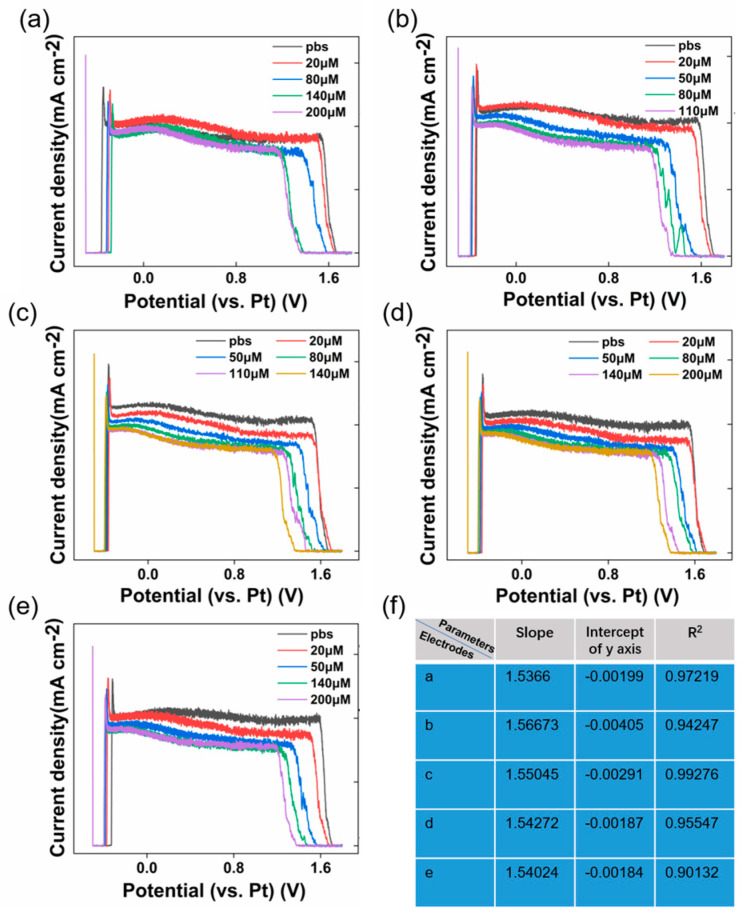
(**a**–**f**) DPV curves of the electrodes (1–5) obtained from Nafion/CB–GO/SPCE in the presence of PBS (0.01 M, pH = 7.2) containing 0–200 μM Tyr. (**b**) Parameters related to the linear fitting curves of the five electrodes.

Figure 4a–f show the DPV diagram of five Nafion/CB-GO/SPCEs prepared by a screen-printing and dispensing process equipped with a multi-channel electrochemical workstation for online Tyr detection. As demonstrated in Figure S1, the terminal of the device was connected to a cylindrical electrolytic cell equipped with built-in multiple electrode columns. According to the steps in Section 2.5, we issued operation commands on the mobile app and set the working mode of the device as DPV and the electrode mode as double electrode. The start potential was −0.5 V, the end potential was 1.8 V, the increase potential was 20 mV, the amplitude was 100 mV, the pulse width was 10 ms, and the pulse period width was 30 ms. In order to facilitate the statistics for the DPV data of each electrode, we connected the upper computer on the laptop computer by USB and used a serial general bus for data transmission. During the experiment, the stability of the environment was ensured by waiting 10 min every time the concentration of Tyr in the solution was changed. Then, the designed TDM program was used to collect the current response data of the five electrodes in the same sequence for each collection round as the Tyr concentration changed.

Using TDM technology, the electrochemical performance of the electrodes could be evaluated more accurately and intuitively. As shown in Figure 4, we found that compared with the DPV curve of the electrode in the pure PBS environment, the presence of Tyr caused a +1.5 V peak in the original DPV diagram line, a negative shift in voltage of the DPV line, and the corresponding current intensity to decrease. Specifically, the voltage data at the extreme point of +1.5 V were selected as the corresponding characteristic value of Tyr, and the DPV curve of the PBS environment was used as the background reference. By fitting the Tyr concentration and characteristic values of the five electrodes collected into linear curves, a linear relationship was observed between them. The specific fitting parameters are shown in Figure 4f. After observation, it was found that for a stable test environment, the successively increasing Tyr powder in the test system led to the same trend of change for the five Nafion/CB-GO/SPCEs in the same batch (i.e., the negative voltage shift of the +1.5 V and the weakened corresponding current). Figure 4f also shows that Nafion/CB-GO/SPCEs prepared by the same process and material had roughly the same linear response curve, and that the Nafion/CB-GO/SPCEs used for Tyr detection had an R^2^ value greater than 0.9. Figure 4f shows the results of fitting the DPV curves of each electrode for Tyr detection, with the independent variable x being the reaction potential values (V) of the DPV curves for different concentrations of Tyr and the dependent variable y being the actual concentration values (μM) of the corresponding Tyr. The linear fitting equations for the five electrodes are shown below:

(1) $$y=1.5366x-0.00199\;R^{2}=0.97129$$

(2) $$y=1.56673x-0.00405\;R^{2}=0.94247$$

(3) $$y=1.55045x-0.00291\;R^{2}=0.99276\;$$

(4) $$y=1.54272x-0.00187\;R^{2}=0.95547\;$$

(5) $$y=1.54024x-0.00184\;R^{2}=0.90132\;$$

The good linearity is attributed to the incorporation of graphene oxide in the combined CB/CP network. On the one hand, the two-dimensional graphene oxide planes play a bridge-like role for the distribution of CB in the continuous channel, and on the other hand, they provide a hydrophilic platform for the riveting of nano-CB particles. The mobile app display is shown in Figure S1.

### 3.2. The Reliability and Specificity of the Nafion/CB-GO/SPCE

**Figure 5:**
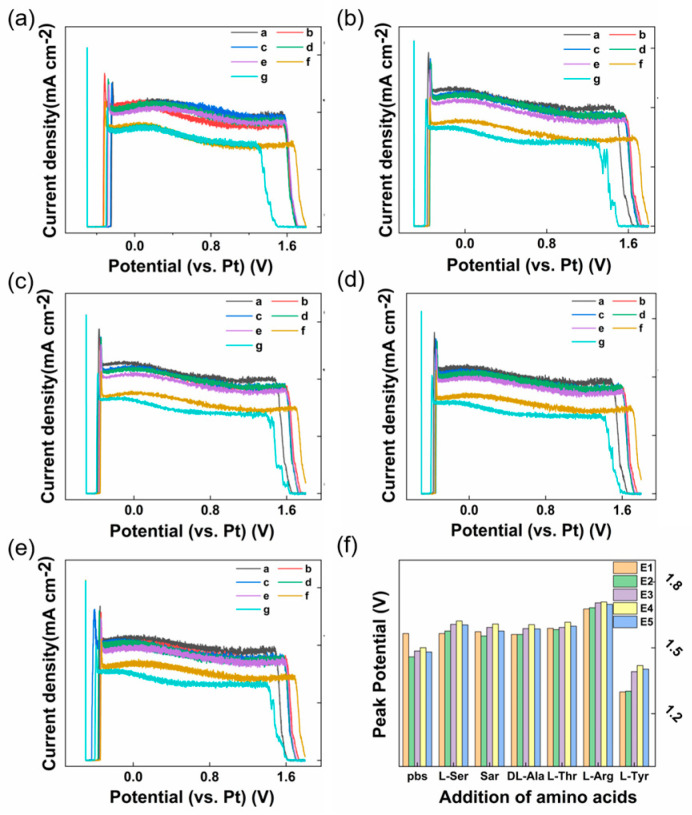
(**a**–**e**) Five electrodes manufactured in the same batch were used for the specificity test of Tyr electrochemical detection by adding 100 μM L-serine, sarcosine, DL-alanine, L-threonine, L-arginine, and Tyr (Line a–g) to the test system (0.01 M PBS, pH = 7.2) in turn. (**f**) Statistical histogram of specific test results.

In order to verify the reliability of the designed multi-channel electrochemical testing system and its specificity for Tyr testing with Nafion/CB-GO/SPCE, five common amino acids were selected as interfering substances. They were L-serine, sarcosine, DL-alanine, L-threonine, and L-arginine. During the experiment, in order to verify that the translation of the negative voltage direction of the wave crest around +1.5 V was a result of the chemical interaction between Tyr and the reaction system, we added 100 μM of the disruptors to the test system in the order of L-serine, sarcosine, DL-alanine, L-threonine, L-arginine, and then we added 100 μM Tyr. The test results are shown in Figure 5a–f. It was found that the disturbance to the test system caused by the addition of the five interferences could be obtained by comparing the DPV diagram of the five electrodes. L-serine, sarcosine, DL-alanine, and L-threonine had no significant electrochemical reaction in the Nafion/CB-GO/SPCE and PBS reaction system. This conclusion was drawn from the nearly coinciding DPV lines in Figure 5a–f. Also, L-arginine was seen around the +1.5 V peak and it was clear that it affected the location of the peak, which also occurred with L-arginine and GO caused by a REDOX reaction. However, the specific detection of Tyr by Nafion/CB-GO/SPCE was not affected. These conclusions can also be seen through a histogram of the specific peak voltage test results, as shown in Figure 5f. Figure 5f shows the true values of the DPV curves for the above five-electrode test system with the sequential addition of 100 μM L-serine, sarcosine, DL-alanine, L-threonine, L-arginine interferents, and Tyr. We can clearly see that compared to the last 100 μM Tyr added to the system, the electrochemical response of the five electrodes to the other interferents differs slightly from the electrochemical response of the electrodes in pure pbs. That proves the good specificity of the synthesized Nafion/CB-GO/SPCE equipped with this multichannel electrochemical workstation for Tyr detection.

### 3.3. Technical Parameters of Multi-Channel Electrochemical Workstation

In order to compare the performance of the multi-channel electrochemical workstation designed with those commonly used in the market, the technical parameters of workstations of related brands were collected and summarized before being compared to the technical parameters of our workstation in Table 2. The multi-channel electrochemical workstation can provide simultaneous test channels of up to 10 electrodes, which is not available in the commercial electrochemical workstations investigated. It also provides a wide voltage range of ±3 V and a current resolution of 6.4 nA. This study provides comparable results at a much lower cost.

### 3.4. Potential Applications

In recent years, the rise of artificial intelligence has triggered technological innovations in various fields. This technology also brings new development opportunities for intelligent electrochemical sensors. Artificial intelligence benefits from the application of big data for model training; thus, it is particularly important for intelligent electrochemical sensors to easily obtain many useful tests. This paper suggests that the use of multiple-electrode electrochemical test platforms could be useful in the field for the analysis of materials. These data could be linked to a cloud-based platform via the Internet of Things to be rapidly utilized by artificial intelligence researchers in the field of electrochemical research, thus greatly reducing the construction time and evaluation cycle of intelligent electrochemical sensor systems.

## 4. Conclusions

In this study, we developed a multi-channel mobile electrochemical test platform. In order to further verify its performance, we used five Nafion/CB-GO/SPCE tablets for online Tyr detection. Nafion/CB-GO/SPCE was used for Tyr preparation through convenient screen-printing and dispensing processes. The electrochemical analog signals from the system were collected by AD through built-in filters and amplifiers and transmitted to a mobile phone app, upper computer, and built-in LCD screen for real-time display. The prepared electrodes and the designed high-throughput electrochemical testing system were characterized in a simulated solution environment. The final test results showed that the constructed multi-channel electrochemical workstation equipped with Nafion/CB-GO/SPCEs for Tyr detection had reliable test accuracy and stability and could effectively save operation time and reduce the accidental error of the system. In this era of big data, being able to obtain a large amount of data quickly and effectively is particularly important. Particularly, when evaluating the reliability of an electrochemical sensor system, achieving a large amount of test data is very important to evaluate and improve the system. Machine learning and other emerging disciplines are driving the demand for electrochemical sensor systems to obtain large sets of test data rapidly. Therefore, our proposed high-throughput electrochemical mobile test platform and the associated intelligent analysis terminal can provide a feasible solution for the next generation of electrochemical equipment for biochemical analysis.

## Figures and Tables

**Table 1:** Unit prices of various ion detection devices and their components.

Components	Cost ($)	Components	Cost ($)
USB chip (CP2102-GMR)	1.8	power module (MAX9934FAUA+)	2.75
USB connector (USB-101)	0.1	CP-032HPJCT-ND (DC_JACK_0.7MM)	0.8
576-1259-1-ND (LDO-MIC5205-3.3YM5-TR)	0.72	576-1259-1-ND (LDO-MIC5205-3.3YM5-TR)	0.36
AD5667RBRMZ-2-ND	9.08	Resistance and capacitance	0.059
AD8608ARUZ	4	PCB	0.5
MAX4643EUA+	1.2	3D-printing module	0.78
MAX4644EUT+T	2.37	LCD	4.31
MK-02A (nRF-52832)	3	Connection terminals	0.05
COMMON MODE FILTER (PDMCAT18107)	0.7	piezoresistor (10D561K)	0.1
Push Button Switch (BTSA)	0.08	power module (HLK-10M05)	3.2
Cost ($)	53.51		

**Table 2:** Comparison between this work and the popular desktop portable electrochemical workstations.

	This Work	Versa STAT 4	CompactStat.h	PalmSens	Interface 1010B
Channels	10	1	1	1	1
Voltage Range	±3 V (67 μV resolution) 40 μV noise	±10 V (6 μV resolution) ±0.2% of reading ± 2 mV	±10 V (0.4 μV resolution) 16/40 nV	±10 V (76.3 μV resolution) ≤0.1% ± 1 mV offset	±12 V (1 μV resolution) ±0.3% of reading ±1 mV
Techniques	OP, CA, CV, DPV, SWV, LSV, NPV	POT, CA, CV, DPV, SWV, OCPT, CPI, LSV	POT, CA, CV, DPV, SWV, LSV, EIS	CA, CV, DPV, SWV, LSV, CC, ZRA, LSP	POT, CA, CV, DPV, SWV
Current Range	1 range: ±180 μA (6.4 nA resolution) 30 nA noise	10 range: ±2 A (120 fA resolution) ±5 pA ± 0.3%	6 range: ±10 A (1.5 p0A resolution) 0.15 fA	9 range: ±10 mA (5 fA resolution) ≤0.1% (at Full Scale Range)	9 range: ±1 A (1.5 pA resolution) ±0.2% of reading, ±0.2% of range 4 nA: <0.5% ± 20 pA
Power source	AC power or USB	AC power	USB	Rechargeable Battery	AC power
Connectivity to Mobile Phones	Wireless connection via Bluetooth Low Energy	NA	WIFI	Wireless connection via Bluetooth	NA
Cost (USD)	53.51	61,880	23,205	9282	38,650

## Data Availability

The sensor data based on multi-channel electrochemical workstation mobile phone is not universal, so it is not disclosed. Interested readers can contact us directly by email.

## Supplementary Materials

The following supporting information can be downloaded at: https://www.mdpi.com/article/10.3390/s23146252/s1, Figure S1: Working diagram of multi-channel electrochemical sensor test platform.; Table S1: Comparison of the analytical parameters obtained for the Bi NPs@GO-MWCNT modified nanocomposite electrode with previously reported modified electrodes. Refs. [28,29,30,31,32,33,34].
