# Supplementary material for: A Time-Division Multiplexing Multi-Channel Micro-Electrochemical Workstation with Carbon-Based Material Electrodes for Online L-Trosine Detection
Source: Sensors (Basel). 2023 Jul 9;23(14):6252. doi: 10.3390/s23146252 (PMC10386381; doi:10.3390/s23146252)
Supplement: Supplementary file 1 [file sensors-23-06252-s001.zip › sensors-2475507-supplementary.pdf]

Supporting information

A time-division multiplexing multi-channel micro-electrochemical workstation with carbon-based material electrodes for online L-tyrosine detection

Qiwen Bao[1], Gang Li[1], Zhengchun Yang[2], Jun Wei[3], Wenbo Cheng[4], Zilian Qu[5], and Ling Lin[1*]

[1]School of Precision Instrument and Optoelectronic Engineering, the State Key Laboratory of Precision Measuring Technology and Instruments, 92 Weijin Road, Tianjin University, Tianjin 300072, China
[2]School of Electrical and Electronic Engineering, Tianjin Key Laboratory of Film Electronic & Communication Devices, Advanced Materials and Printed Electronics Center, Tianjin University of Technology, Tianjin 300384, China
[3]School of Materials Science and Engineering, Harbin Institute of Technology, Shenzhen 518055, China
[45]Suzhou Institute of Biomedical Engineering and Technology, Chinese Academy of Sciences, Suzhou 215163, China
[5]Beijing Informat Technol Coll, Beijing 100015, China
*Corresponding author; email: linling@tju.edu.c

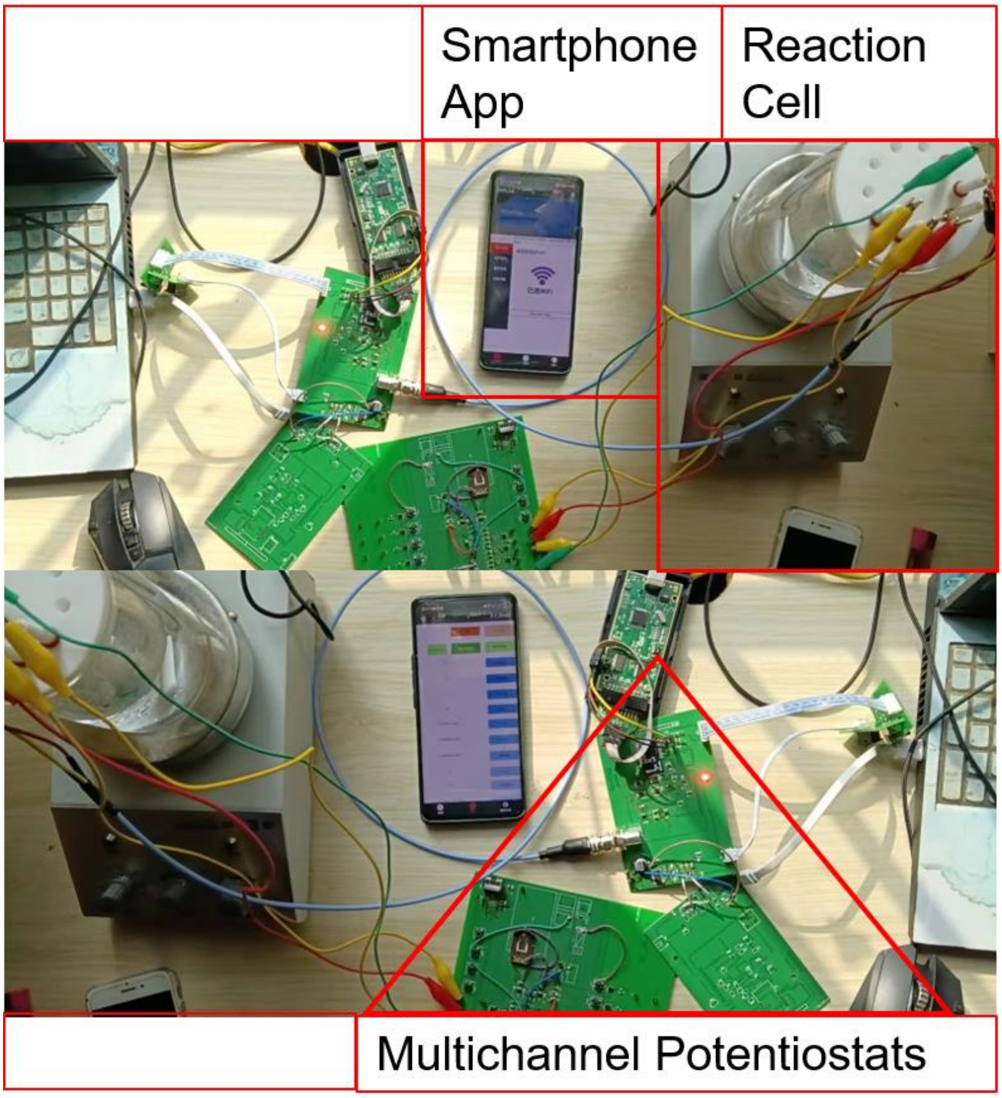

Figure S1 Working diagram of multi-channel electrochemical sensor test platform.

Table S1

Comparison of the analytical parameters obtained for the Bi NPs@GO-MWCNT modified nanocomposite electrode with previously reported modified electrodes.

Electrode	LOD (nM)	Sensitivity	Liner Range (μM)	Ref
Macroporous carbon embedded with sulfato-beta-cyclodextrin (MPC-SCD) hybrid	260	73.02 μA/mM cm^2	1–500	*[1]*
Screen Printed Carbon Electrode (SPCE) with	0.05	–	0.1-60	*[2]*

Graphene Oxide-COOH/Chitosan (GO-COOH/Chitosan) electro-deposition				
Graphene Oxide-epsilon-MnO2 Microspheres/Chitosan Modified Activated Glassy Carbon Electrode	*8.3*	–	0.02–20	*[3]*
Cysterici acid - modified glassy carbon electrode	*1100*	–	3.5-96	*[4]*
Poly L-methionine/Electrochemically Reduced Graphene Oxide Composite Film Modified Glassy Carbon Electrode (poly(L-Met)/ERGO/GCE))	0.27		1–70	*[5]*
Crown Ether/Ionic Liquid Crystal-Carbon Nanotubes	*1.42*	–	0.01-60	*[6]*
Poly (Diphenylamine) Modified Electrode	*20000*		20–1000	*[7]*
Carbon-graphene oxide/screen-printed carbon electrodes (CB-GO/SPCE)	*20×10^3*		20-200	This work

References

1. Zhao, J.; Cong, L.R.; Ding, Z.Y.; Zhu, X.J.; Zhang, Y.F.; Li, S.H.; Liu, J.; Chen, X.; Hou, H.B.; Fan, Z.; et al. Enantioselective electrochemical sensor of tyrosine isomers based on macroporous carbon embedded with sulfato-beta-Cyclodextrin. *Microchemical Journal* **2020**, *159*, doi:10.1016/j.microc.2020.105469.
2. Fooladi, E.; Razavizadeh, B.M.; Noori, M.; Kakooei, S. Application of carboxylic acid-functionalized of graphene oxide for electrochemical simultaneous determination of tryptophan and tyrosine in milk. *Sn Applied Sciences* **2020**, *2*, doi:10.1007/s42452-020-2332-0.
3. Wang, S.Q.; Zhai, H.Y.; Chen, Z.G.; Wang, H.H.; Tan, X.C.; Sun, G.H.; Zhou, Q. Constructing a Sensitive Electrochemical Sensor for Tyrosine Based on Graphene Oxide-epsilon-MnO2

Microspheres/Chitosan Modified Activated Glassy Carbon Electrode. *Journal of the Electrochemical Society* **2017**, *164*, B758-B766, doi:10.1149/2.0571714jes.

4. Hassanvand, Z.; Jalali, F. Simultaneous determination of L-DOPA, L-tyrosine and uric acid by cysteic acid - modified glassy carbon electrode. *Materials Science and Engineering C-Materials for Biological Applications* **2019**, *98*, 496-502, doi:10.1016/j.msec.2018.12.131.
5. Swathy, S.; Mathew, M.R.; Kumar, K.G. Poly L- methionine/Electrochemically Reduced Graphene Oxide Composite Film Modified Glassy Carbon Electrode for the Simultaneous Determination of 5-hydroxyindole Acetic Acid and Tyrosine. *Journal of the Electrochemical Society* **2022**, *169*, doi:10.1149/1945-7111/ac8ad3.
6. Atta, N.F.; Galal, A.; Ahmed, Y.M. Highly Conductive Crown Ether/Ionic Liquid Crystal-Carbon Nanotubes Composite Based Electrochemical Sensor for Chiral Recognition of Tyrosine Enantiomers. *Journal of the Electrochemical Society* **2019**, *166*, B623-B630, doi:10.1149/2.0771908jes.
7. Krishnan, R.G.; Saraswathyamma, B. Simultaneous Resolution and Electrochemical Quantification of Tyrosine and Tryptophan at a Poly (Diphenylamine) Modified Electrode. *Journal of the Electrochemical Society* **2021**, *168*, doi:10.1149/1945-7111/abe1dd.
